# A potential pathogenic mutation of *LAMA4* in a Chinese family with dilated cardiomyopathy and conduction system disease

**DOI:** 10.1097/MD.0000000000040875

**Published:** 2024-12-13

**Authors:** Huaigen Wang, Ronghui Zhao, Jiaojiao Wang, Xiu Han, Kaifeng Li, Yafeng Gao, Ya Wang, Aiqun Ma, Tingzhong Wang, Yuan Du

**Affiliations:** a Department of Cardiovascular Medicine, The First Affiliated Hospital of Xi’an Jiaotong University, Xi’an, Shaanxi, China; b Department of Anaesthesiology and Surgery, The First Affiliated Hospital of Xian Jiaotong University, Xi’an, Shaanxi, China; c Department of Cardiovascular Medicine, Luonan County Hospital, Xi’an, Shaanxi, China; d Shaanxi Key Laboratory of Molecular Cardiology (Xi’an Jiaotong University), Xi’an, Shaanxi, China; e Shaanxi International Science & Technology Cooperation Base for Cardiovascular Precision Medicine, Xi’an, Shaanxi, China.

**Keywords:** conduction system disease, dilated cardiomyopathy, G218R, *LAMA4*, next generation sequencing

## Abstract

Dilated cardiomyopathy (DCM) is characterized by ventricular dilation and poor systolic function. Approximately half of idiopathic DCM cases are assigned to genetic causes in familial or apparently sporadic cases, and more than 50 genes are reported to cause DCM. However, genetic basis of most DCM patients still keeps unknown and require further study. Clinical data, family histories, and blood samples were collected from the proband and family members in a Chinese family presenting with DCM and conduction system disease. A genetic analysis was performed using next generation sequencing (NGS). Bioinformatic analysis was performed to predict the pathogenic consequence of gene mutation. A missense heterozygous mutation c.652G > A (p.G218R) in *Laminin Subunit Alpha-4* (*LAMA4*) gene was identified in proband and his 2 brothers with relevant clinical symptoms. Individuals without carrying this mutation in this family had no symptoms or cardiac structural abnormality related to DCM or conduction system disease. The p.G218R mutation is located in a conservative area within the laminin epidermal growth factor (EGF)-like domain of *LAMA4* with uncertain significance in ClinVar archive. Bioinformatic analysis predicted p.G218R mutation as deleterious and pathogenic damaging in DCM patients. Our results reported a potential pathogenic mutation associated with DCM, which may provide further insight into genetic contributions of *LAMA4* gene mutations to DCM phenotypes.

## 1. Introduction

Dilated cardiomyopathy (DCM) is a condition characterized by ventricular dilation and systolic dysfunction.^[1]^ At least half of portion of heart failure is diagnosed as idiopathic DCM with unknown cause. Approximately 50% idiopathic DCM cases are assigned to genetic causes in familial or apparently sporadic cases,^[2]^ and more than 50 genes are reported to cause DCM.^[3]^ These genes encode components of the sarcomere, desmosome, cytoskeleton, nuclear lamina and mitochondria, and calcium-handling proteins.^[3]^ Pathogenic variants in the *TTN* gene, which codes for TITIN located in the sarcomere, account for nearly 30% to 35% of familial DCM cases. The second most prevalent gene in familial DCM is *LMNA* (which encodes LAMIN A/C), be responsible for nearly 5% to 10% of all familial DCM cases.^[4]^ We have previously identified a novel insertion mutation (nucleotide 1526insA, amino acid T510Y) in *LMNA* carried by a young female in a family with dilated cardiomyopathy, conduction system disease and sudden cardiac death.^[5]^ However, genetic basis of most DCM patients keeps unknown and require further study.

Genetic studies focusing on the main genes associated with DCM have mainly used conventional Sanger sequencing, despite the practical difficulties of keeping up with the ever-increasing number of disease-associated genes and test requests.^[6]^ In recent years, next generation sequencing (NGS) has emerged as a revolutionary technology that enables the generation of huge quantities of genetic data.^[7]^ This plethora of information has triggered the development of bioinformatic tools to help interpret potential causality.^[8]^

In the present study, using a NGS assay combined with clinical assessments, we identified a potential mutant in *Laminin Subunit Alpha-4* (*LAMA4*), p.G218R (c.652G > A), in a Chinese family diagnosed as DCM and conduction system disease. Our findings provide further insight into genetic contributions of *LAMA4* gene mutations to DCM phenotypes.

## 2. Methods

### 2.1. Clinical assessment

Written informed consent was obtained from all family members included in the study. DCM diagnosis was made based on the World Health Organization 1995 criteria for the classification of cardiomyopathies. Detailed clinical evaluation, including medical history, physical examination, serum CK, 12-lead electrocardiogram (ECG) and transthoracic echocardiography, was performed in the proband and all relatives. One hundred unrelated blood donors with normal ECGs and echocardiography served as controls. This study was approved by the Ethics Committee of the First Affiliated Hospital of Xi’an Jiaotong University (Xi’an, Shaanxi, China) and conforms to the principles outlined in the Declaration of Helsinki.

### 2.2. Samples

Blood samples of the proband and his family members were collected. RNA-free high-molecular-weight DNA was prepared using a blood DNA Extraction Kit (TIANGEN [DP318-03(200)], Beijing, China). The quality and concentration of genomic DNA were assessed by agarose gel electrophoresis and using a NanoDrop spectrophotometer.

### 2.3. Next generation sequencing and analysis

To prepare sequencing libraries, 1000 ng genomic DNA was fragmented to an average size of 250 bp. The DNA fragments were ligated to 8 bp barcoded sequencing adaptors and then hybridized to a customer-made exome panel focused on genes related to cardiovascular disease (NimbleGen, Roche). This NGS gene panel not only included cardiomyopathy-related genes, but included all genes known to be associated with cardiovascular diseases (in total 1876 genes, Table S1, Supplemental Digital Content, http://links.lww.com/MD/O161). The quality and quantity of adaptor-ligated fragments were measured using a fragment analyzer (Advance Analytical, Ankeny, Iowa) and qPCR. Purified sequencing libraries were pooled together and massively parallel sequenced on the Illumina HiSeq X ten platform to produce an average of 2.5 Gb per sample with an average sequencing depth of approximately 150×. These data were then aligned to the hg19 genome using NextGene (V2.3.4, Softgenetics, State College, Pennsylvania), an alignment method that is similar to BLAST methodology to align sequence reads to the reference. NextGene uses a preloaded index alignment algorithm that employs a suffix array that is represented by the Burrows-Wheeler Transform (BWT). All the mutations were compared with databases, such as dbSNP, ExAC or the 1000 Genomes Project. All identified mutations were subject to Sanger sequencing confirmation using an ABI3130 Genetic analyzer (Applied Biosystems, Waltham, Massachusetts).

### 2.4. Conservation analysis and Bioinformatic prediction

The multiple sequence alignment software, ClustalX- 2.1-win (Science Foundation Ireland, Dublin, Ireland), was used to analyze the conservation of *LAMA4* p.Gly218 in *Homo sapiens* and other species. The Polyphen-2 (v2.2.8), PROVEAN (v1.1.3), MutationTaster2021 and Combined Annotation-Dependent Depletion (CADD, v1.6) algorithms were used to predict the pathogenicity of detected mutation. The reference protein ID of LAMA4 was “P00748” and the Ensembl transcript ID was “ENST00000253496.” This mutation was further classified in accordance with the American College of Medical Genetics and Genomics (ACMG) Standards and Guidelines.^[9]^

## 3. Results

### 3.1. Clinical features of the proband

**Figure 1.**
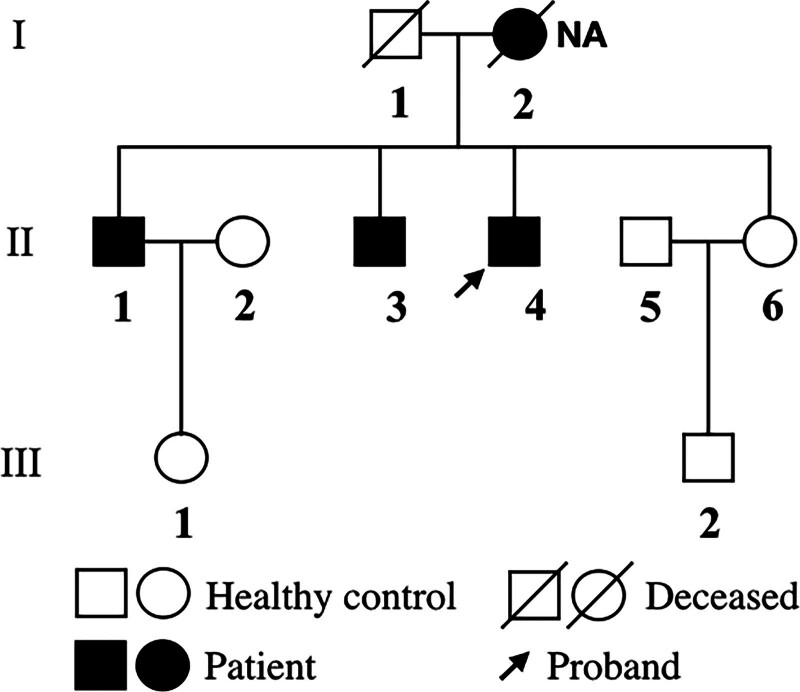
Pedigree structure of the family. Family members are identified by generations and numbers. Square, male family member; circle, female member. NA = DNA sample is not available.

**Figure 2.**
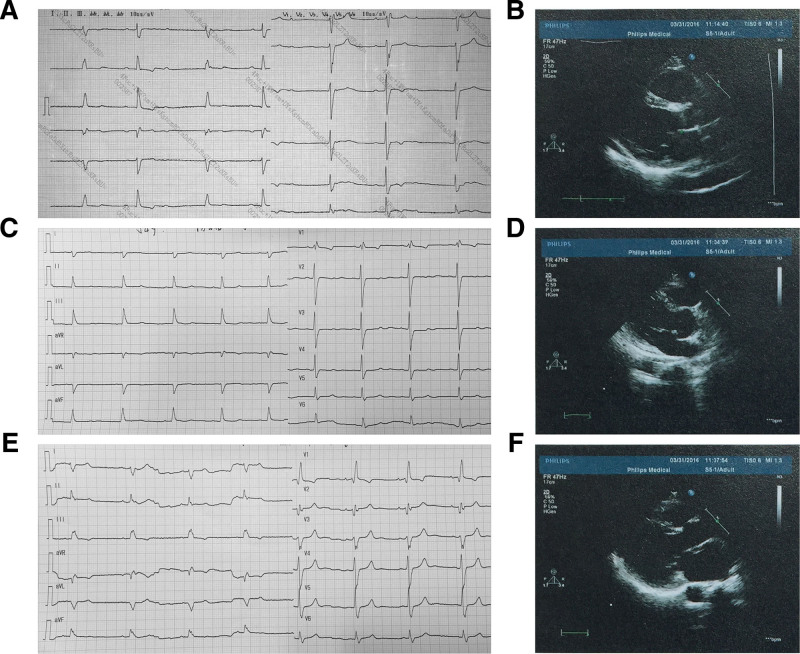
The ECG records and transthoracic echocardiography data the proband and his brothers. ECG of II-4 (A), II-1 (C) and II-3 (E), and the transthoracic echocardiography data of II-4 (B), II-1 (D) and II-3 (F). ECG = electrocardiogram.

The proband (II-4, Fig. 1) presented to our hospital in 2013 having experienced with fatigue and shortness of breath for 3 years. His echocardiogram revealed a moderately dilated left ventricle (LV) (left ventricular end diastolic diameter, LVDD: 64 mm) with normal systolic function (left ventricular ejection fraction, LVEF: 54%). His electrocardiogram showed high-degree atrioventricular block (AVB) and right bundle branch block (RBBB, Fig. 2A). Coronary angiography was normal. A permanent dual chamber pacemaker was implanted. The proband was followed-up and no further episodes of ventricular tachycardia (VT) were identified. In 2016, the patient experienced progressive dyspnea, abdominal distention and edema of the lower limbs. His echocardiography (Fig. 2B) showed LV size was greatly dilated (LVDD: 70 mm) with significantly depressed systolic function (LVEF: 35%). His NT-proBNP was 7671 pg/mL (normal range, 0–125pg/mL). During the proband’s clinical visit, we were informed that his relatives had similar symptoms.

### 3.2. Family pedigree

The proband’s mother (I-2, Fig. 1) died of heart failure at the age of 70. Unfortunately, her blood sample were not available to us. The proband’s father (I-1, Fig. 1) died of a cerebrovascular event at the age of 67 without any clinical symptoms of heart failure. The proband’s first brother (II-1, Fig. 1) had mild symptoms of heart failure. He also showed sinus bradycardia and RBBB (Fig. 2C). His LV size was dilated (LVDD: 60 mm) but systolic function was normal (LVEF: 65%) (Fig. 2D). The clinical history of the second brother (II-3, Fig. 1) was very similar to the proband’s. His clinical manifestations were shortness of breath and edema. His electrocardiogram showed sinus bradycardia and RBBB (Fig. 2E), and his echocardiogram indicated LV dilation (LVDD: 76 mm) and dysfunction (LVEF: 37%) (Fig. 2F). The proband and his second brother were unmarried and had no offspring. The proband’s 2 brothers had no history of taking β-blockers or digitalis, and the sinus bradycardia was thought unrelated to medicines therapies. The remaining 5 family members (including II-2, II-5, II-6, III-1, and III-2, Fig. 1) were asymptomatic and had normal echocardiogram and ECGs results. There were no manifestations of myasthenia or other skeletal muscle diseases in any family members, and the levels of creatine kinase(CK) were all in normal range during our investigations.

### 3.3. Mutation identification

To identify the genetic basis of DCM and conduction system disease suffered by this family, a custom-made NGS panel consisting of 1876 genes previously associated with cardiomyopathies and related striated muscle disorders was used (Table S1, Supplemental Digital Content, http://links.lww.com/MD/O161). Genetic sequencing was performed in the proband using this NGS panel.

**Figure 3.**
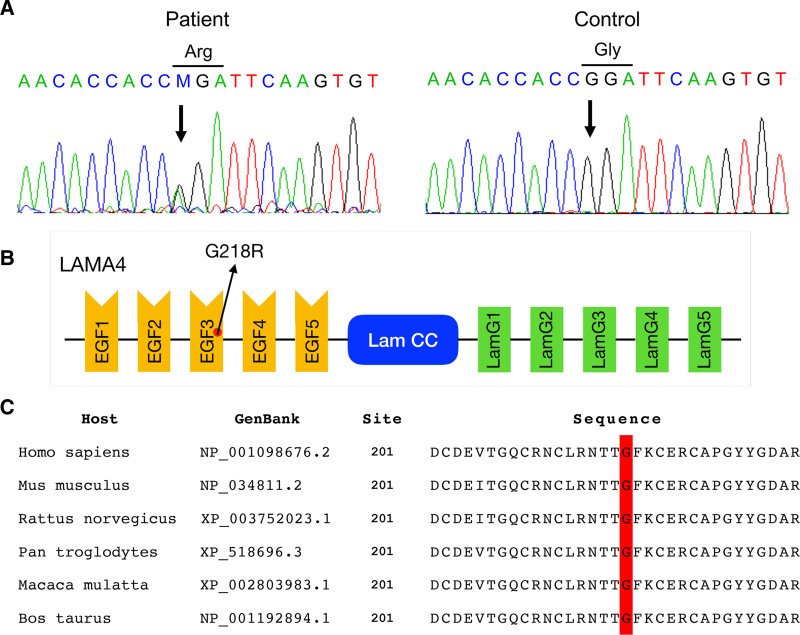
A missense mutation of *LAMA4* identified in DCM and conduction system disease. (A) Electropherogram of the sequence encompassing the heterozygous G to M (G/A mixed) transition at nucleotide 652 (Gly218Arg, p.G218R). (B) A diagram illustrating the LAMA4 protein. Arrow indicates the location of p.G218R mutation. LAMA4 consists of 5 EGF (epidermal growth factor) repeats, Lam CC (laminin coiled coil), and 5 LamG (laminin-α chain carboxyterminal globular) domains. (C) Evolutionary conservation of Gly218 in orthologs shown in red. DCM = dilated cardiomyopathy, LAMA4 = laminin subunit alpha-4.

A missense mutation c.652G > A was found in *LAMA4* (RefSeq: NM_001105206.3), corresponding to a nonsynonymous amino acid change from glycine to arginine at position 218 (p.G218R, Fig. 3A). This mutation affected the well-conserved glycine residue at position 218 in *LAMA4* in multiple species, which is an epidermal growth factor (EGF) repeat (Fig. 3B). This evolutionary conserved site is located within the laminin EGF-like domain, which binds to basement membrane proteins (Fig. 3C).

Next, the above c.652G > A mutation in *LAMA4* was sequenced in the other family members. This mutation was confirmed in the 2 other affected family members (II-1, II-3, Fig. 1) and was not seen in the proband’s 5 unaffected relatives (Fig. 1, Table 1). By evaluating the genotype and echocardiogram parameters and clinical features relationship, data in Table 1 demonstrating significant differences in conduction system function and heart function of the mutation carriers compared with the noncarriers within the family. This further indicates that *LAMA4* c.652G > A underlies the DCM and conduction system disease in this family. Family genetic testing for the LAMA4 c.652G > A (p.Gly218Arg) variant has provided supporting-level evidence (PP1) for segregation with the disease in the proband (II-4, Fig. 1) and his 2 brothers (II-1, II-3, Fig. 1), taking into account that this variant is absent in large population cohorts of gnomAD, thereby meeting the moderate-level criterion PM2.

**Table 1:** Clinical characteristics of the family members.

ID	Gender	Age	NYHA	ECG	HR (bpm)	Echocardiography			*LAMA4* Mutation	Comment
						LVDD (mm)	LAD (mm)	LVEF (%)		
I-1	M	67	−	−	−	−	−		−	Died of cerebrovascular event
I-2	F	70	−	−	−	−	−		−	Died of HF
II-1	M	54	II	Sinus bradycardia, RBBB	53	60	32	65	c.652G > A	Slight symptoms of HF
II-3	M	50	III	Sinus bradycardia, RBBB	51	76	39	37	c.652G > A	HF
II-4 Proband	M	46	IV	High-degree AVB, RBBB	41	70	42	35	c.652G > A	HF
II-6	F	41	I	Normal	63	48	29	71	No	Asymptomatic
III-1	F	27	I	Normal	61	51	−	66	No	Asymptomatic
III-2	M	8	I	Normal	74	36	−	60	No	Asymptomatic

Abbreviations: AVB = atrioventricular block, bpm = beat per minute, ECG = electrocardiogram, HF = heart failure, HR = heart rate, LAD = left atrial diameter, LVDD = left ventricular end diastolic diameter, LVEF = left ventricular ejection fraction, NYHA = New York heart association functional class, RBBB = right bundle branch block.

### 3.4. Pathogenicity prediction

The c.652G > A (p.G218R) mutation in *LAMA4* has been submitted in ExAC and ClinVar database. However, evidence for *LAMA4*-DCM relationship is limited and the clinical significance of this p.G218R mutation is unclear. Our bioinformatic analysis results using the PolyPhen-2, PROVEAN, and CADD algorithms confirmed that the p.G218R mutation is deleterious and pathogenic damaging (Table 2). MutationTaster2021 further predicted this mutation as disease causing (Table 2). According to the ACMG criteria, the c.652G > A (p.G218R) mutation in *LAMA4* were classified as likely pathogenic.

**Table 2:** Prediction results of p.G218R in *LAMA4*.

Tools	Score	Prediction results	Meaning
PolyPhen-2	1.000	Most probably damaging	Evaluated as 0.000 (most probably benign) to 1.000 (most probably damaging)
PROVEAN (v1.1.3)	−4.034	Deleterious	Score ≤ −2.5 (deleterious), >−2.5 (neutral)
CADD	31	Deleterious	Score ≥ 20 as more deleterious
MutationTaster2021	0.9999	Disease causing	A probability close to 1 indicates a high security of prediction

## 4. Discussion

Our report describes a Chinese family with DCM characterized by heart failure and conduction system disease. Genetic testing identified a missense heterozygous mutation c.652G > A (p.G218R) in the *LAMA4* gene with an autosomal dominant pattern of inheritance, which might to be a potential pathogenic mutation associated with DCM.

The *LAMA4* gene is located on chromosome 6q21 and includes 39 exons. It encodes laminin subunit alpha-4, which is a component of extracellular matrix (ECM) proteins laminin-8 and -9.^[10]^ Laminins serve as a scaffold for the attachment of other ECM proteins, and they also impact cell differentiation and phenotype maintenance by participating in cell signaling.^[11]^ *LAMA4* is localized mainly in basement membranes of blood vessels of the adult heart and in the peripheral sarcolemma of cardiomyocytes. The abundance of *LAMA4* transcripts in the heart suggests an important role of this protein in cardiovascular development and function.^[12]^

However, *LAMA4* mutations associated with DCM are uncommon and its estimated contribution to patients is approximately 1%.^[13,14]^ To date, there have only a few reports concerning *LAMA4* mutations in DCM patients. In hundreds of Caucasian patients with severe DCM, Knoll et al found 1 individual carrying a *LAMA4* c.3217C > T (p.R1073X) mutation and 2 individuals carrying a *LAMA4* c.2828C > T (p.P943L) mutation.^[15]^ The 2 DCM patients with the c.2828C > T mutation were sporadic and without a genetic relationship. These 2 *LAMA4* mutations are located in the laminin G domain (LamG).

The laminin alpha-4 integrin–integrin-linked kinase pathway is central in conveying signals from the outside to the inside of a cell, and regulating endothelial cell attachment, migration, and proliferation. Mutations in this system affect endothelial cell and cardiomyocyte survival and lead to cardiomyopathy. In *LAMA4* knockout mice hearts, endothelial defects (endothelial wall thinning and ruptures), irregularity of capillaries and enlargement of the space between myocytes and adjacent capillary vessels were observed, which might cause insufficient oxygen supply to the heart. These mutant mice gradually developed cardiac hypertrophy and cardiac dysfunction.^[16]^ The *LAMA4* c.3217C > T (p.R1073X) and c.2828C > T (p.P943L) mutations in DCM patients mentioned above are located in the integrin-interacting domain of *LAMA4*. These missense mutations cause loss of integrin-binding capacity and lead to a significant decrease in endothelial cell adherence. This leads to a strong reduction in endothelial cell numbers and to a disruption of the interaction between endothelial cells and cardiomyocytes and the extracellular matrix, which ultimately results in cardiomyopathy and heart failure.^[15]^

Here we identified a missense mutation in *LAMA4*, c.652G > A (p.G218R), located in the laminin-type EGF-like domain (EGF_Lam) in exon 6 that is different location from previously reported c.3217C > T (p.R1073X) and c.2828C > T (p.P943L) mutations. This mutation has been submitted into ClinVar archive with uncertain significance by 2 groups recently.^[17]^ The mechanism of how this mutation interferes with the function of laminin subunit alpha-4, leading to dilation of the cardiac chambers and conduction system disease are not yet clear. Therefore, understanding how this mutation lead to DCM phenotype are key for genetic diagnosis and management of those patients carrying this mutation.

Our bioinformatic analysis results suggested that the p.G218R mutation is deleterious and pathogenic damaging. Mutation Taster further predicted this mutation as disease causing. These results raised the possibility that *LAMA4* c.652G > A (p.G218R) mutant may caused dysfunction of conveying signals from the outside to the inside of a cell, affect endothelial cell function, eventually leading to DCM. However, bioinformatic analysis cannot reflect the real pathology of the *LAMA4* c.652G > A (p.G218R) mutation on the cardiac myocytes.

The inaccessibility of human heart tissue is a barrier for the pathogenesis study of genetic heart diseases, the emergence of patient-derived induced pluripotent stem cells (iPSCs) and genome editing tools such as CRISPR/Cas9 strategy may provide an exciting new approach to understand disease mechanisms underpinning inherited heart diseases.^[18,19]^ In the future, we will further evaluate the pathogenicity of *LAMA4* c.652G > A (p.G218R) mutation using CRISPR/Cas9 gene editing method to create mutant mice or using patient-specific iPSCs -derived cardiomyocytes.

Numerous factors can determine penetrance and expressivity, including age/sex differences and genetic modulators.^[20]^ The penetrance of the *LAMA4* mutation in this family is high; all 3 mutation carriers manifested with varying degrees of a dilated ventricle and conduction system disease. The symptoms of heart failure were mildest in the proband’s first brother. Importantly, none of the individuals without the genetic mutation showed any symptom or cardiac structural abnormality related to DCM.

In the present study, the proband’s LV dilated (LVDD from 64 to 70 mm) and the heart function decreased markedly (LVEF from 54% to 35%) during the 3 years after his initial visit. We think the *LAMA4* mutation is the primary cause of DCM progression. The permanent pacemaker probably played a precipitating role in asynchronism of left and right ventricles, which affected heart function. The implantation of cardiac resynchronization therapy defibrillators was suggested, but not carried out for the proband (II-4) or his second brother (II-3).

Conventional genetic sequencing studies investigate a small number of disease-associated genes. NGS enables the detailed screening of a large number of genes in cardiac gene panels (i.e., >100 genes) at relatively low cost and using a limited amount of DNA. It is important to note that our custom-made NGS panel not only included currently known DCM-associated genes but also genes related to inherited channelopathies^[21]^ because we have previously demonstrated that mutation delQKP 1507 to 1509 in the cardiac sodium channel encoding gene, *SCN5a*, is associated with the expanding phenotypic spectrum of DCM, conduction disorder, long QT syndrome type 3 and sudden youth death.^[22]^

## 5. Conclusions

The present study reported a potential pathogenic mutation in *LAMA4* in a Chinese family presenting with DCM and conduction system disease. This provides further insight into genetic contributions to DCM pathology. Furthermore, genetic testing may allow a better clinical management of these patients with a wide range of clinical presentation and can help to improve the care of patients with DCM.

## Acknowledgments

We thank the highly qualified native English-speaking editors at Edanz Group China for editing the English.

## Author contributions

**Conceptualization:** Huaigen Wang, Aiqun Ma, Tingzhong Wang, Yuan Du.

**Data curation:** Huaigen Wang, Kaifeng Li.

**Formal analysis:** Yafeng Gao, Ya Wang.

**Funding acquisition:** Ya Wang, Aiqun Ma, Tingzhong Wang, Yuan Du.

**Investigation:** Ronghui Zhao, Xiu Han.

**Methodology:** Ronghui Zhao, Xiu Han, Kaifeng Li, Yafeng Gao.

**Project administration:** Jiaojiao Wang.

**Resources:** Jiaojiao Wang, Kaifeng Li.

**Software:** Jiaojiao Wang.

**Writing—original draft:** Huaigen Wang.

**Writing—review & editing:** Aiqun Ma, Yuan Du.

## Supplementary Material


