# Supplementary material for: A potential pathogenic mutation of LAMA4 in a Chinese family with dilated cardiomyopathy and conduction system disease
Source: Medicine (Baltimore). 2024 Dec 13;103(50):e40875. doi: 10.1097/MD.0000000000040875 (PMC11651427; doi:10.1097/MD.0000000000040875)
Supplement: Supplementary file 1 [file medi-103-e40875-s001.docx]

**Supplementary Table S1. Detailed information of NGS gene panel for cardiovascular diseases (1876 genes).**

| **Cardiovascular diseases** | **No.** | **Gene name** |
| --- | --- | --- |
| Congenital heart disease | 275 | ACTC1, ABCC6, ABCC9, ABCD4, ACTA2, ACTB, ACTN2, ACVR2B, ADAMTS10, ADAMTSL2, ADK, AFF4, ALDH1A2, ALX3, AMER1, ANKS6, ARHGAP31, ARL2BP, ARMC4, ASXL1, ATIC, ATRX. B3GALT6, B3GAT3, B3GLCT, BBS2, BCOR, BRAF, C21ORF59, CACNA1D, CAV3, CBL, CCBE1, CCDC103, CCDC114, CCDC151, CCDC39, CCDC40, CD96, CEP120, CEP290, CEP57, CFAP53, CFC1, CHD7, CHRM3, CHST14, CHST3, CITED2, CKAP2L, COMT, COQ4, COX7B, CRB2, CREBBP, CRELD1, CTCF, DDX11, DHCR24, DHCR7, DIS3L2, DLL4, DNAAF1, DNAAF2, DNAAF3, DNAAF5, DNAH11, DNAH5, DNAI1, DNAI2, DNAL1, DNMT3A, DPM1, DSG1, DTNA, DYNC2H1, DYX1C1, ECE1, ECHS1, EDNRA, EFTUD2, ELN, EOGT, ERBB3, ESCO2, EVC, FADD, FBLN5, FBN1, FBN2, FGFR2, FIG4, FKBP14, FKTN, FLNA, FLNB, FOXC1, FOXC2, FOXF1, FOXH1, FTO, G6PC3, GATA4, GATA6, GBA, GDF1, GJA1, GLI3, GPC3, GPC6, HAND1, HCCS, HES7, HGD, HIBCH, HPGD, HRAS, HYLS1, IDUA, IFIH1, IFT122, IFT172, IFT43, IGF2, IGFBP7, INVS, IRX5, JAG1, KANSL1, KAT6A, KAT6B, KCNH1, KDM6A, KIAA0196, KMT2D, KRAS, LBR, LMNA, LONP1, LRP2, LRP5, LRRC6, LTBP4, LZTFL1, LZTR1, MAP2K1, MAP2K2, MED12, MED13L, MEGF8, MEOX1, MGAT2, MGP, MKS1, MPLKIP, MRPS16, MTFMT, MYCN, MYH11, MYH6, MYH7, MYLK2, NAA10, NEK1, NEK8, NEXN, NF1, NFIX, NIPBL, NKX2-5, NKX2-6, NODAL, NOTCH1, NOTCH2, NOTCH3, NPHP3, NR2F2, NRAS, NSD1, PALB2, PEX1, PEX16, PHGDH, PIEZO2, PIGA, PIGL, PIGN, PIGT, PIK3CA, PIK3R2, POLR1A, PQBP1, PRKAG2, PRRX1, PTF1A, PTPN11, RAB23, RAF1, RARB, RBM10, RBM8A, RBP4, RIT1, RNU4ATAC, ROR2, RPL11, RPL15, RPL26, RPL35A, RPS19, RPS26, SALL1, SALL4, SCN1B, SEMA3E, SETBP1, SF3B4, SGOL1, SH3BP2, SHANK3, SHOC2, SLC19A2, SLC29A3, SLC2A10, SMAD3, SMAD4, SMAD6, SMN1, SNIP1, SNRPB, SOS1, SOS2, SOX2, SPAG1, SPECC1L, SRCAP, STAMBP, STK4, STRA6, TAB2, TAF2, TALDO1, TBX1, TBX20, TBX3, TBX5, TCTN3, TFAP2B, TFAP2B, TGDS, TGFB2, TGFB3, TGFBR1, TGFBR2, THOC6, TLL1, TMCO1, TP63, TSFM, TTC37, TTC7A, TTC8, TXNL4A, UBR1, UMPS, VIPAS39, VPS33B, WDPCP, WDR60, ZEB2, ZFPM2, ZIC3, ZMPSTE24, ZMYND10, ZNF423 |
| Atrial septal defect (ASD) | 100 | ACTC1, ABCD4, ACTN2, ADAMTSL2, ADK, AMER1, ARHGAP31, ASXL1, ATIC, B3GALT6, B3GAT3, BBS2, BCOR, BRAF, CCBE1, CEP57, CHD7, CHST14, CITED2, COX7B, CREBBP, CRELD1, CTCF, DHCR7, DNMT3A, DTNA, ECE1, EFTUD2, ESCO2, EVC, FBN1, FBN2, FGFR2, FKTN, FLNB, FOXC1, FOXF1, G6PC3, GATA4, GATA6, GJA1, HCCS, HRAS, IRX5, JAG1, KANSL1, KAT6B, KDM6A, KIAA0196, KMT2D, KRAS, MAP2K1, MAP2K2, MED12, MYH6, MYH7, NAA10, NEK1, NEXN, NF1, NFIX, NKX2-5, NODAL, NPHP3, NR2F2, NSD1, PHGDH, PIGA, PIGN, PIK3R2, PQBP1, PTF1A, PTPN11, RAB23, RIT1, RNU4ATAC, RPS19, SALL4, SEMA3E, SETBP1, SHOC2, SLC19A2, SLC29A3, SRCAP, STAMBP, STK4, STRA6, TBX20, TBX5, TGFB3, TGFBR1, TGFBR2, TLL1, TMCO1, UBR1, UMPS, VIPAS39, VPS33B, ZEB2, ZMPSTE24 |
| Ventricular septal defect (VSD) | 137 | ACVR2B, ADAMTS10, AMER1, ARHGAP31, ASXL1, ATRX, B3GALT6, B3GLCT, BCOR, BRAF, CACNA1D, CCBE1, CD96, CEP290, CEP57, CHD7, CHST3, CITED2, CKAP2L, COMT, CRB2, CREBBP, CRELD1, DDX11, DHCR7, DLL4, DSG1, DTNA, ECE1, ECHS1, EFTUD2, EOGT, ERBB3, ESCO2, FADD, FBN1, FBN2, FGFR2, FIG4, FLNB, FOXC2, FOXH1, FTO, GATA4, GATA6, GDF1, GJA1, GLI3, GPC3, GPC6, HAND1, HCCS, HRAS, HYLS1, IFT172, JAG1, KANSL1, KAT6A, KAT6B, KIAA0196, KMT2D, KRAS, LBR, LONP1, LRP2, LRP5, MED12, MED13L, MEOX1, MGAT2, MGP, MPLKIP, MTFMT, NAA10, NEK1, NIPBL, NKX2-5, NKX2-6, NODAL, NOTCH1, NOTCH2, NR2F2, NSD1, PALB2, PEX1, PEX16, PHGDH, PIGL, PIK3CA, PIK3R2, PQBP1, PTPN11, RAB23, RAF1, RARB, RBM8A, RIT1, ROR2, RPL15, RPL35A, RPS19, RPS26, SALL1, SALL4, SEMA3E, SF3B4, SHANK3, SHOC2, SLC19A2, SLC29A3, SMAD6, SMN1, SNRPB, SOS1, SOX2, SPECC1L, STAMBP, STRA6, TALDO1, TBX1, TBX3, TBX5, TCTN3, TGDS, TGFB3, TP63, TTC37, TTC7A, TXNL4A, UBR1, UMPS, VIPAS39, VPS33B, WDR60, ZEB2, ZFPM2, ZIC3 |
| Patent ductus arteriosus (PDA) | 81 | ABCC9, ACTA2, ACTB, ADAMTS10, AFF4, AMER1, ANKS6, BCOR, CD96, CEP120, CHD7, CHRM3, COQ4, CREBBP, CTCF, DHCR24, DHCR7, DPM1, DTNA, ECE1, EOGT, ESCO2, FBN1, FBN2, FKBP14, FLNA, FOXC1, FOXC2, FOXF1, FTO, G6PC3, GATA6, GLI3, GPC3, HPGD, IGF2, KAT6A, KCNH1, LMNA, MEGF8, MKS1, MRPS16, MYCN, MYH11, NFIX, NKX2-5, NOTCH2, NOTCH3, NPHP3, NSD1, PEX1, PHGDH, PIGN, PIGT, POLR1A, PTPN11, RAB23, RBP4, RPS26, SEMA3E, SF3B4, SHANK3, SMAD3, SMAD4, SOX2, SPECC1L, STRA6, TALDO1, TBX1, TBX5, TFAP2B, TGFBR1, TGFBR2, THOC6, TMCO1, TP63, TSFM, ZEB2, ZIC3, ZMPSTE24, NR2F2 |
| Tetralogy of fallot (TOF) | 32 | ALDH1A2, ALX3, ARHGAP31, BRAF, CHD7, COX7B, DDX11, FIG4, FOXC2, GATA4, GATA6, GDF1, HIBCH, JAG1, KIAA0196, NKX2-5, NKX2-6, NR2F2, PIGL, PQBP1, RAB23, RBM10, RBM8A, RNU4ATAC, RPL11, SALL1, SEMA3E, SF3B4, STRA6, TBX1, TTC37, ZFPM2 |
| Pulmonary stenosis | 58 | ACVR2B, ADAMTSL2, ADK, ALDH1A2, ALX3, ANKS6, ARHGAP31, BRAF, CFAP53, CHD7, COX7B, DDX11, DTNA, ELN, FBN1, FIG4, FKTN, FOXC2, GATA4, GATA6, GDF1, GPC6, HIBCH, IFT43, IGFBP7, JAG1, KIAA0196, KRAS, LTBP4, MAP2K1, MAP2K2, MGP, NAA10, NKX2-5, NKX2-6, NOTCH2, NR2F2, NRAS, PIGL, PQBP1, PTPN11, RAB23, RAF1, RBM10, RBM8A, RNU4ATAC, RPL11, SALL1, SEMA3E, SF3B4, SLC2A10, SOS1, STRA6, TBX1, TTC37, ZEB2, ZFPM2, ZIC3 |
| Aortic stenosis | 22 | ACTB, ADAMTSL2, ANKS6, CAV3, CBL, CEP57, ELN, ESCO2, FBLN5, GBA, IDUA, IFIH1, KIAA0196, MYH7, MYLK2, NKX2-5, NPHP3, NR2F2, PTPN11, SCN1B, SLC2A10, WDPCP |
| Coarctation of the aorta | 35 | ABCD4, ADK, CEP57, DHCR7, GATA6, GDF1, GLI3, GLI3, HRAS, JAG1, KDM6A, KMT2D, KRAS, LZTR1, MKS1, MYH7, NKX2-5, NKX2-6, NR2F2, NRAS, PTPN11, RBM8A, RNU4ATAC, RPS19, SMAD4, SMAD6, SOS2, SPECC1L, SRCAP, STRA6, TALDO1, TBX1, TBX20, TGDS, WDPCP |
| Bicuspid aortic valve | 24 | ABCC9, ACTB, B3GALT6, B3GAT3, BBS2, CBL, EDNRA, FBN2, FLNA, IFT122, KANSL1, MYH11, MYH7, NAA10, NOTCH1, RPL26, SGOL1, SMAD6, SNIP1, TAB2, TAF2, TGFB2, TGFBR1, TGFBR2 |
| Dextrocardia | 43 | ACVR2B, ANKS6, ARL2BP, ARMC4, BCOR, C21ORF59, CCDC103, CCDC114, CCDC151, CCDC39, CCDC40, CFAP53, CFC1, CRELD1, DNAAF1, DNAAF2, DNAAF3, DNAAF5, DNAH11, DNAH5, DNAI1, DNAI2, DNAL1, DYX1C1, GDF1, HES7, INVS, LRRC6, LZTFL1, MEGF8, NEK8, NODAL, PIEZO2, PQBP1, PRRX1, SLC19A2, SPAG1, TGDS, TTC8, UBR1, ZIC3, ZMYND10, ZNF423 |
| Mitral stenosis | 8 | ABCC6, ADAMTSL2, CHST3, FBN1, GBA, HGD, IFIH1, LZTR1 |
| Mitral regurgitation | 28 | ADAMTS10, AGA, B3GALT6, CBL, CHST3, COL1A2, CRYAB, DCHS1, DTNA, ELN, FBLN5, FBN1, FBN2, FLNA, G6PC3, IDUA, IRX5, LMNA, MYPN, PDSS1, PIK3R2, POLG, PRDM16, RPS6KA3, SGOL1, SMAD3, SMAD4, TGFB3 |
| Mitral valve prolapse | 31 | ABCC6, B3GAT3, BCOR, CBS, COL1A1, COL1A2, COL2A1, COL3A1, COL5A1, COL5A2, DCHS1, FBN1, FBN2, FLNA, FMR1, HRAS, KRAS, MFAP5, POLG, SKI, SLC29A3, SMAD3, SMAD4, TGFB2, TGFBR1, TGFBR2, TNXB, VPS13B, VWF, XYLT2, ZNF469 |
| Aortic stenosis | 30 | ACTB, ADAMTS10, ADAMTSL2, ANKS6, BCOR, CBL, CHST3, CYP24A1, DDX58, ELN, ESCO2, FBLN5, FBN1, GBA, GLB1, GNPTG, IDUA, IFIH1, KIAA0196, LTBP2, NOTCH1, NPHP3, NR2F2, SCN1B, SLC2A10, SMAD4, SNIP1, VWF, HGD, NKX2-5 |
| Congenital double outlet of the right ventricle | 9 | BCOR, CHD7, GATA6, GDF1, KIAA0196, NKX2-5, NKX2-6, SEMA3E, TBX1 |
| Hypoplastic left heart syndrome | 6 | NR2F2, KIAA0196, GJA1, NKX2-5, DTNA, TBX5 |
| Transposition of great arteries | 14 | GDF1, MEGF8, RAB23, GATA6, NODAL, NKX2-5, FKTN, CFAP53, PIGL, PHGDH, TBX1, NKX2-6, MED13L, GPC3 |
| Noonan syndrome | 13 | BRAF, CBL, DYNC2H1, KRAS, MAP3K8, NF1, NRAS, PTPN11, RAF1, RIT1, SH3BP2, SHOC2, SOS1 |
| Dilated cardiomyopathy (DCM) | 103 | ABCC9, ABCC9, ACAD8, ACAD9, ACTA1, ACTC1, ACTN2, ADCY5, ALMS1, ANKRD1, BAG3, BBS2, BOLA3, CAV3, CHKB, CHRM2, CPT2, CRYAB, CSRP3, CTF1, DES, DMD, DNAJC19, DOLK, DPM3, DSC2, DSG2, DSP, EMD, EPG5, ERBB3, ETFA, ETFB, ETFDH, EYA4, FHL1, FHL2, FKRP, FKTN, FLNC, FLT1, GATAD1, GBE1, GLB1, HADH, HADHA, HADHB, ILK, ISL1, JUP, LAMA4, LAMP2, LDB3, LMNA, MGME1, MURC, MYBPC3, MYH6, MYH7, MYPN, NDUFB11, NEBL, NEXN, PDCD1, PDLIM3, PGM1, PKP2, PLN, POLG, PRDM16, PSEN1, PSEN2, RAF1, RBCK1, RBM20, RYR1, SCN5A, SDHA, SDHAF1, SDHD, SEPN1, SGCB, SGCD, SPEG, SYNE1, SYNE2, SYNM, TAZ, TCAP, TMPO, TNNC1, TNNI3, TNNI3K, TNNT2, TPM1, TPM3, TSFM, TTN, TXNRD2, UBR1, VCL, XK, XPNPEP3 |
| Hypertrophic cardiomyopathy (HCM) | 168 | AARS2, ABCC9, ACAD9, ACADVL, ACTC1, ACTN2, AGK, AGPAT2, ANKRD1, ANKS6, ANO5, APOPT1, ATP5E, ATPAF2, BAG3, BCS1L, BRAF, BSCL2, CALR3, CAPN3, CASQ2, CAV3, COA5, COA6, COQ2, COQ4, COX10, COX14, COX15, COX20, COX6B1, CRYAB, CSRP3, DES, DLD, DSG2, DYSF, ELAC2, EYA4, FAH, FASTKD2, FBXL4, FHL1, FHL2, FKRP, FKTN, FLNC, FOXRED1, FTO, FXN, GAA, GLA, GLB1, GNPTAB, GNS, HADH, HGSNAT, HRAS, HSD17B10, INS-IGF2, JPH2, KLF1, KLF10, KRAS, LAMA4, LAMP2, LDB3, LIAS, LMNA, LRPPRC, MAP2K1, MAP2K2, MLYCD, MRPL3, MRPL44, MRPS22, MTO1, MYBPC1, MYBPC3, MYH6, MYH7, MYL2, MYL3, MYLK2, MYO6, MYOM1, MYOT, MYOZ2, MYPN, NAGLU, NDUFA1, NDUFA10, NDUFA11, NDUFA2, NDUFAF1, NDUFAF2, NDUFAF3, NDUFAF4, NDUFAF5, NDUFAF6, NDUFB3, NDUFB9, NDUFS1, NDUFS2, NDUFS3, NDUFS4, NDUFS6, NDUFS7, NDUFS8, NDUFV1, NDUFV2, NEXN, NRAS, NUBPL, PDHA1, PDHB, PDLIM3, PDSS2, PET100, PLN, POMGNT1, POMT1, POMT2, PRKAG2, PSEN1, PSEN2, PTPN11, RAF1, RBM20, RIT1, RYR2, SCO1, SCO2, SDHA, SDHAF1, SDHD, SGCA, SGCB, SGCD, SGCG, SGSH, SHOC2, SLC22A5, SLC25A3, SLC25A4, SMC1A, SOS1, SRI, SURF1, TACO1, TAZ, TCAP, TFB1M, TMEM126A, TMEM70, TNNC1, TNNI3, TNNT2, TPM1, TRIM32, TRIM54, TRIM63, TSFM, TTN, TTR, VCL, XPNPEP3, YARS2 |
| Arrhythmogenic right ventricular cardiomyopathy (ARVC) | 26 | CTNNA3, DES, DNAJC19, DSC2, DSG2, DSP, FOXH1, GATA4, GATA6, GDF1, GJA1, HAND1, JUP, LMNA, MED13L, NKX2-5, NKX2-6, NOTCH1, PKP2, PLN, RYR2, SMAD6, TGFB3, TMEM43, TTN, ZFPM2 |
| Restrictive cardiomyopathy | 10 | ABCC6, ACTC1, DES, GLA, MYH7, MYL3, PDGFRA, PIGT, TNNI3, TNNT2 |
| Left ventricular noncompaction | 11 | DTNA, MIB1, LDB3, MYH7, TNNT2, PRDM16, TPM1, MYBPC3, ACTN2, ACTC1, CASQ2 |
| Long QT syndrome (LQTS) | 44 | ADAMTS10, AKAP9, ANK2, ATP1B1, CACNA1C, CALM1, CAV3, DNAJC19, FBN1, FOXH1, GATA4, GATA6, GDF1, GJA1, GMPPB, GYS1, GYS2, HAND1, KCNA5, KCNE1, KCNE2, KCNE3, KCNH2, KCNJ2, KCNJ5, KCNQ1, KCNQ1OT1, LIG3, LITAF, MECP2, MED13L, NKX2-5, NKX2-6, NOS1AP, NOTCH1, PLN, PTRF, SCN4B, SCN5A, SMAD6, SNTA1, TRPM4, ZFPM2, CALM2 |
| Short QT syndrome | 6 | CACNA1C, CACNA2D1, CACNB2, KCNH2, KCNJ2, KCNQ1 |
| Brugada syndrome | 17 | CACNA1C, CACNA2D1, CACNB2, GPD1L, HCN4, KCND3, KCNE3, KCNJ16, KCNJ8, RANGRF, SCN10A, SCN1B, SCN2B, SCN3B, SCN5A, TRPM4, SLMAP, KCNE5 |
| Catecholamine sensitivity ventricular tachycardia | 5 | CALM1, CALM3, CASQ2, RYR2, TRDN |
| Early repolarization syndrome | 7 | KCNJ8, ABCC9, SCN5A, CACNA1C, CACNB2B, CACNA2D1, KCND2 |
| Atrial fibrillation | 54 | ABCC9, ACTN2, ANK2, CACNA1C, CACNB2, CSRP3, CYP11B2, CYP4F2, DMPK, DPP6, DTNA, GATAD1, GJA5, HCN4, KCNA5, KCND3, KCNE1, KCNE1L, KCNE2, KCNE3, KCNH2, KCNJ2, KCNJ5, KCNQ1, LMNA, MFAP5, MYH6, MYOZ2, MYPN, NEXN, NKX2-5, NKX2-6, NPPA, NUP155, PITX2, PLN, PRKAG2, PTRF, RYR2, SCN10A, SCN1B, SCN2B, SCN4B, SCN5A, SGOL1, SMAD3, TAB2, TBX5, TLL1, TMEM43, TNNI3, TNNI3K, TNNT2, ZFHX3 |
| Ventricular fibrillation | 11 | DSP, GPD1L, KCNE1, KCNE2, KCNE3, KCNH2, KCNQ1, NDUFB11, SCN1B, SCN5A, SNTA1 |
| Ventricular tachycardia | 22 | ABCC9, CALM1, CASQ2, CSRP3, CTNNA3, DSG2, DSP, GNAI2, HCN4, JPH2, JUP, MYL2, MYOZ2, NAA10, NDUFB11, PRKAG2, RYR2, SCN5A, SLC25A20, TMEM43, TPM1, TRDN |
| Supraventricular tachycardia | 43 | ABCC9, ACTN2, ANK2, CACNA1C, CACNB2, CLIC2, CSRP3, DMPK, DTNA, EMD, GATAD1, GJA5, HCN4, KCNA5, KCNE2, KCNJ2, KCNJ5, KCNQ1, LMNA, MFAP5, MYOZ2, MYPN, NAA10, NEXN, NKX2-5, NPPA, NUP155, PLN, PRKAG2, PTRF, RYR2, SCN1B, SCN2B, SCN3B, SCN4B, SCN5A, SGOL1, SMAD3, TLL1, TMEM43, TNNI3, TNNI3K, TNNT2 |
| Atrial flutter | 8 | DMPK, LMNA, SGOL1, SCN3B, CLIC2, SCN5A, TNNI3K, NUP155 |
| Cardiac conduction block | 23 | RNASEH1, DMPK, MYOZ2, SGOL1, POMT2, GPD1L, HCN4, GJA1, TRPM4, TNNI3K, GJA5, GYG1, KCNJ5, SLC25A20, SCN1B, PTPN11, ACTN2, EMD, SCN4B, SCN5A, AGXT, PRKAG2, CTNNA3 |
| Primary atrial arrhythmia | 42 | MYOZ2, GATAD1, CACNA1C, KCNE2, KCNQ1, NKX2-5, ABCC9, MFAP5, TNNI3K, GJA5, NPPA, CACNB2, PTRF, TMEM43, ACTN2, EMD, KCNA5, PRKAG2, ANK2, NUP155, DMPK, LMNA, TNNI3, SGOL1, TNNT2, HCN4, PLN, CLIC2, NEXN, DTNA, KCNJ2, CSRP3, KCNJ5, SCN1B, TLL1, RYR2, SCN2B, MYPN, SMAD3, SCN3B, SCN4B, SCN5A |
| Supraventricular arrhythmia | 44 | COL4A1, MYOZ2, NAA10, GATAD1, CACNA1C, KCNE2, KCNQ1, NKX2-5, ABCC9, MFAP5, TNNI3K, GJA5, NPPA, CACNB2, PTRF, TMEM43, ACTN2, EMD, KCNA5, PRKAG2, ANK2, NUP155, DMPK, LMNA, TNNI3, SGOL1, TNNT2, HCN4, PLN, CLIC2, NEXN, DTNA, KCNJ2, CSRP3, KCNJ5, SCN1B, TLL1, RYR2, SCN2B, MYPN, SMAD3, SCN3B, SCN4B, SCN5A |
| Arrhythmia | 190 | ABCC9, ABCC9, ACADVL, ACTC1, ACTN2, ADAMTS10, AGXT, AKAP9, ANK2, ATP1B1, BANF1, BRAT1, C10ORF2, CACNA1B, CACNA1C, CACNA1D, CACNA2D1, CACNB2, CACNB2B, CALM1, CALM2, CALM3, CASQ2, CAV3, CLIC2, CNBP, COL4A1, COQ4, CPOX, CPT1A, CPT2, CREBBP, CSRP3, CTNNA3, CYP11B2, CYP4F2, DES, DMD, DMPK, DNAJC19, DPP6, DSC2, DSG2, DSP, DST, DTNA, EFEMP2, EMD, ERCC6, ERCC8, EYA4, FBN1, FBP1, FBXL4, FHL1, FMO3, FOXC2, FOXH1, GAA, GATA4, GATA6, GATAD1, GDF1, GJA1, GJA5, GLA, GLUL, GMPPB, GNAI2, GPC3, GPD1L, GTPBP3, GYG1, GYS1, GYS2, HAND1, HCCS, HCN4, HFE, HMBS, HRAS, IKBKAP, ISCU, JPH2, JUP, KCNA5, KCND2, KCND3, KCNE1, KCNE1L, KCNE2, KCNE3, KCNE5, KCNH2, KCNJ16, KCNJ2, KCNJ5, KCNJ8, KCNK3, KCNQ1, KCNQ1OT1, KIF1B, KYNU, LAMP2, LDB3, LIG3, LITAF, LMNA, MECP2, MED13L, MFAP5, MGME1, MTFMT, MTO1, MYH6, MYH7, MYL2, MYLK2, MYOZ2, MYPN, NAA10, NDUFB11, NEXN, NKX2-5, NKX2-6, NOS1AP, NOTCH1, NPPA, NUP155, PAX8, PGM1, PHYH, PITX2, PKP2, PLN, POLG2, POMT2, PPOX, PRDM16, PRKAG2, PSMB8, PTPN11, PTRF, RAF1, RANGRF, RBM20, RET, RNASEH1, RYR1, RYR2, SCN10A, SCN1B, SCN2B, SCN3B, SCN4B, SCN5A, SCN9A, SDHB, SDHC, SDHD, SGOL1, SLC19A2, SLC25A20, SLMAP, SMAD3, SMAD6, SNTA1, SYNE2, TAB2, TAZ, TBX5, TCAP, TGFB3, TLL1, TMEM43, TMEM70, TNNI3, TNNI3K, TNNT2, TPM1, TRDN, TRPM4, TSC1, TSC2, TSHR, TSPYL1, TTN, VHL, ZFHX3, ZFPM2 |
| Primary aldosteronism | 14 | SCNN1A, BSND, SCNN1B, CLCNKA, CLCNKB, SCNN1G, KCNJ1, CYP11A1, CYP11B1, KCNJ5, NR3C2, SLC26A3, KCNJ10, SLC12A1 |
| Pseudohypoaldosteronism | 8 | CUL3, KLHL3, NR3C2, SCNN1A, SCNN1B, SCNN1G, WNK1, WNK4 |
| Aortic dissection | 9 | ACTA2, COL3A1, FBN1, MYH11, MYLK, SMAD3, TGFB2, TGFBR1, TGFBR2 |
| Ehlers-Danlos Syndrome | 14 | ADAMTS2, B3GALT6, B4GALT7, CHST14, COL1A1, COL1A2, COL3A1, COL5A1, COL5A2, DSE, FKBP14, PLOD1, SLC39A13, TNXB |
| Thoracic aortic aneurysm and dissection. | 7 | TGFBR1, TGFBR2, MYH11, ACTA2, FBN1, MYLK, SMAD3 |
| Hypercholesterolemia | 18 | ABCG5, ABCG8, APOB, APOE, APTX, CAV1, EPHX2, JAG1, LDLR, LDLRAP1, LIPA, LPL, OCRL, PCSK9, PHKA2, RAI1, SLC25A13, TTPA |
| Pulmonary artery hypertension | 41 | ABCD4, ACTA2, ACVRL1, ARHGAP31, ATP5A1, BANF1, BMPR1B, BMPR2, CACNA1D, CAV1, CHST3, COL1A1, COL1A2, COX7B, EIF2AK4, FBN1, FGFR3, FIG4, FLNA, FOXF1, G6PC3, GATA6, GBA, GJA1, IDUA, KCNK3, KRT18, KRT8, LIFR, LIPA, NFIX, NFU1, NOTCH1, PAM16, PDSS1, SARS2, SFTPA2, SFTPB, SLC37A4, SMAD9, SPECC1L |
| Marfan's syndrome | 18 | ACTA2, CBS, COL11A1, COL11A2, COL2A1, COL3A1, COL5A1, COL5A2, COL9A1, COL9A2, FBN1, FBN2, MYH11, MYLK, PLOD, SMAD3, TGFBR1, TGFBR2 |
| Aortectasia | 25 | ACTA2, B3GAT3, C12ORF57, COL1A1, COL5A1, COL5A2, EFEMP2, FBLN5, FBN1, FBN2, FLNB, HGD, KANSL1, KCNH1, MED12, MFAP5, MYH11, MYLK, PRKG1, SKI, SMAD3, SMAD4, TAB2, TGFBR1, TGFBR2 |
| Congestive heart failure | 77 | ABCC6, ABCC9, ACAD9, ACTC1, ADAMTSL2, ADCY5, ALMS1, APOA1, ATP5A1, BAG3, CAV3, CEP19, CLIC2, COL1A1, COL1A2, CSRP3, DES, DMD, DNAJC19, DSP, DTNA, ELAC2, ENG, ENPP1, EPG5, EYA4, FBN1, FGFR3, FLNA, FXN, GATAD1, GJA1, GLA, GLB1, GNPTAB, GTPBP3, HADHA, HADHB, HFE, IDS, IFIH1, JUP, KCNJ5, KIF1B, LDB3, LMNA, MYH7, MYLK2, MYPN, PHYH, PLN, PLOD1, PRKAG2, PRKAR1A, PSEN1, PSEN2, PSMB8, RAB3GAP2, RBM20, RET, RPS19, SCN5A, SDHB, SDHD, SGCD, SLC17A5, SLC22A5, TAZ, TF, TMPO, TNNI3, TNNI3K, TNNT2, TPI1, TPM1, TRIM37, VHL |
| Sudden cardiac death (SCD) | 282 | AARS2, ABCC9, ACAD8, ACAD9, ACADVL, ACTA1, ACTC1, ACTN2, ADAMTS10, ADCY5, AGK, AGPAT2, AKAP9, ALMS1, ANK2, ANKRD1, ANKS6, ANO5, APOPT1, ATP1B1, ATP5E, ATPAF2, BAG3, BBS2, BCS1L, BOLA3, BRAF, BSCL2, CACNA1C, CACNA2D1, CACNB2, CALM1, CALM2, CALM3, CALR3, CAPN3, CASQ2, CAV3, CHKB, CHRM2, COA5, COA6, COQ2, COQ4, COX10, COX14, COX15, COX20, COX6B1, CPT2, CRYAB, CSRP3, CTF1, CTNNA3, DES, DLD, DMD, DNAJC19, DOLK, DPM3, DSC2, DSG2, DSP, DTNA, DYSF, ELAC2, EMD, EPG5, ERBB3, ETFA, ETFB, ETFDH, EYA4, FAH, FASTKD2, FBN1, FBXL4, FHL1, FHL2, FKRP, FKTN, FLNC, FLT1, FOXH1, FOXRED1, FTO, FXN, GAA, GATA4, GATA6, GATAD1, GBE1, GDF1, GJA1, GLA, GLB1, GMPPB, GNAI2, GNPTAB, GNS, GPD1L, GYS1, GYS2, HADH, HADHA, HADHB, HAND1, HCN4, HGSNAT, HRAS, HSD17B10, ILK, INS-IGF2, ISL1, JPH2, JUP, KCNA5, KCND3, KCNE1, KCNE2, KCNE3, KCNE5, KCNH2, KCNJ16, KCNJ2, KCNJ5, KCNJ8, KCNQ1, KCNQ1OT1, KLF1, KLF10, KRAS, LAMA4, LAMP2, LDB3, LIAS, LIG3, LITAF, LMNA, LRPPRC, MAP2K1, MAP2K2, MECP2, MED13L, MGME1, MLYCD, MRPL3, MRPL44, MRPS22, MTO1, MURC, MYBPC1, MYBPC3, MYH6, MYH7, MYL2, MYL3, MYLK2, MYO6, MYOM1, MYOT, MYOZ2, MYPN, NAGLU, NDUFA1, NDUFA10, NDUFA11, NDUFA2, NDUFAF1, NDUFAF2, NDUFAF3, NDUFAF4, NDUFAF5, NDUFAF6, NDUFB11, NDUFB3, NDUFB9, NDUFS1, NDUFS2, NDUFS3, NDUFS4, NDUFS6, NDUFS7, NDUFS8, NDUFV1, NDUFV2, NEBL, NEXN, NKX2-5, NKX2-6, NOS1AP, NOTCH1, NRAS, NUBPL, PDCD1, PDHA1, PDHB, PDLIM3, PDSS2, PET100, PGM1, PKP2, PLN, POLG, POMGNT1, POMT1, POMT2, PRDM16, PRKAG2, PSEN1, PSEN2, PTPN11, PTRF, RAF1, RANGRF, RBCK1, RBM20, RIT1, RYR1, RYR2, SCN10A, SCN1B, SCN2B, SCN3B, SCN4B, SCN5A, SCO1, SCO2, SDHA, SDHAF1, SDHD, SEPN1, SGCA, SGCB, SGCD, SGCG, SGSH, SHOC2, SLC22A5, SLC25A3, SLC25A4, SLMAP, SMAD6, SMC1A, SNTA1, SOS1, SPEG, SRI, SURF1, SYNE1, SYNE2, SYNM, TACO1, TAZ, TCAP, TFB1M, TGFB3, TMEM126A, TMEM43, TMEM70, TMPO, TNNC1, TNNI3, TNNI3K, TNNT2, TPM1, TPM3, TRDN, TRIM32, TRIM54, TRIM63, TRPM4, TSFM, TTN, TTR, TXNRD2, UBR1, VCL, XK, XPNPEP3, YARS2, ZFPM2 |
